# Accuracy of anthropometric-based predictive equations for tracking fat mass over a competitive season in elite female soccer players: a validation study

**DOI:** 10.1186/s13102-025-01115-4

**Published:** 2025-04-03

**Authors:** Giulia Baroncini, Francesco Campa, Priscilla Castellani Tarabini, Alberto Sala, Lorenzo Boldrini, Stefano Mazzoni, Antonio Paoli

**Affiliations:** 1 Milan Lab Department, AC Milan, Milan, Italy; 2 Department of Biomedical Sciences, University of Padua, Padua, Italy

**Keywords:** Anthropometry, Body composition, DXA, Fat mass, Soccer, Female athletes

## Abstract

**Background and aims:**

Body fat is a key body composition parameter monitored in soccer. Identifying reliable alternatives to laboratory techniques for assessing body fat during the competitive period is essential. This study aimed to evaluate the cross-sectional and longitudinal validity of anthropometric prediction equations in elite female soccer players.

**Methods:**

Eighteen female soccer players (age: 26.6 [3.8] years; height: 168 [6.3] cm; body mass: 64.1 [7.4] kg; body mass index: 22.7 [1.9] kg/m²) from an Italian Serie A team were assessed at four time points during a competitive season. Fat mass was estimated using anthropometric equations by Evans and Warner and compared to dual-energy X-ray absorptiometry (DXA), which served as the reference method.

**Results:**

Cross-sectional agreement analysis revealed a bias of -4.5% with Warner’s equation, while Evans’s equation showed no bias compared to DXA, with coefficient of determination (R²) values of 0.69 and 0.70, respectively. Both methods showed a negative association (Evans: *r* = -0.53, Warner: *r* = -0.63) when the difference between the values and the mean with DXA were correlated. Longitudinal agreement analysis showed no significant differences in fat mass changes between the anthropometric equations and DXA, with R² values ranging from 0.68 to 0.83. The 95% limits of agreement between the methods for individual changes in fat mass ranged from − 3.3 to 3.2%. Furthermore, no significant changes (*p* > 0.05) in fat mass were observed over the season with any method.

**Conclusions:**

At the group level, Evans’s equation provides valid estimates of fat mass, whereas it may overestimate values in players with low body fat and underestimate them in those with high fat mass. The Warner equation showed the same trend as Evans at the individual level, also resulting in poor accuracy at the group level. Despite this, both anthropometric equations are valid alternatives to DXA for monitoring fat mass changes during the season, with Evans’s equation showing superior overall performance.

**Supplementary Information:**

The online version contains supplementary material available at 10.1186/s13102-025-01115-4.

## Introduction

Soccer performance is influenced by a variety of physical features, in addition to the technical and tactical skills specific to the game. Among the factors, it has been suggested that body composition could play an important role, influencing overall performance in high-level soccer athletes [1–3]. Body composition refers to the different components that make up body mass, such as fat, muscle, bones, and fluids [4]. Some of these components, such as those related to bone structure (e.g., heights, lengths, and breadths) cannot be substantially modified through training or nutrition in adult players [5, 6]. These characteristics are often related to the playing positions, with goalkeepers typically having the tallest statures [7]. Other factors, like fat, muscle, and water levels, are modifiable and can be maintained within optimal ranges, regardless of playing position [5]. Indeed, one hallmark of elite soccer players is maintaining low body fat levels, as excess fat negatively impacts aerobic capacity and mobility [8]. As a result, one of the primary goals during the preparatory phase and transition periods is to closely monitor changes in body fat [9]. Additionally, tracking changes in body composition remains a key component of an athlete’s fitness assessment throughout the playing season [10, 11].

Among the most accurate and commonly used methods to monitor body fat in soccer research is dual-energy X-ray absorptiometry (DXA), an indirect imaging technique based on the different pattern of X-rays absorption by human body tissues [12]. DXA enables the quantification of body mass at the molecular level, including both visceral and subcutaneous lipids, collectively termed fat mass [13]. However, its use is limited to laboratory settings or organizations with substantial financial resources due to its high cost [14]. Consequently, research has focused on developing valid, more affordable, and less invasive alternatives suitable for practical applications. Among these are doubly indirect methods that utilize prediction equations designed to yield results comparable to those obtained through DXA or other reference techniques [15, 16]. These prediction equations are based on raw anthropometric or bioelectrical parameters, and their use is widespread among nutritionists, strength coaches, and researchers. Although the literature is abundant with prediction equations, their validity is closely dependent on the context in which they are applied [17, 18], akin to a suit that fits differently depending on the individual wearing it.

Researches have extensively focused on the development and validation of anthropometric equations to estimate fat mass, identifying specific and accurate formulas for soccer players [10, 19–21]. However, to our knowledge, no study has examined the accuracy of the equations currently available for female soccer players, resulting in a disparity in the procedures used to quantify body fat in this specific athletic population [22–25]. Recent discussions in sports science emphasize the growing need for gender balance in research, particularly due to the historical underrepresentation of female athletes [26]. Most studies have focused on male populations, limiting our understanding of how women respond to training, nutrition, and changes in body composition [27]. Calls for more gender-inclusive research have grown stronger, urging targeted studies to address these gaps and ensure evidence-based recommendations for female athletes [27].

Assessing whether the existing anthropometric equations are valid and determining which ones perform best would improve the monitoring of body fat during the competitive season, allowing for the optimization of nutritional and training interventions [5]. Furthermore, this investigation would help clarify whether the development of new soccer-specific equations is necessary in the future. Therefore, the aim of this study was to evaluate the accuracy of anthropometric equations by identifying the most suitable ones for female soccer players and assessing their validity over the course of a competitive season. We hypothesized that certain equations would be better suited than others, yielding values closely aligned with those obtained through DXA measurements.

## Materials and methods

### Study design

The present investigation was designed as an observational, one-group, four-time study. The sample size was determined for convenience, including all athletes who were part of the selected sports team. An a priori statistical power analysis was conducted to determine the sample size using G*Power statistical software. Consequently, given the study design and an effect size falling between “small” and “medium” the estimated required sample size was 18 subjects. The initial assessment time point (T1) took place in the pre-season period (August 2023). The second assessment (T2) took place in the first midseason phase (November 2023). The third assessment (T3) was completed during the second part of the season (February 2024). The final time point (T4) took place at the end of the season (May 2024). At each time point, participants were assessed using DXA and anthropometry.

### Subjects

A total of 18 female soccer players (age: 26.6 [3.8] y, height: 168 [6.3] cm, body mass: 64.1 [7.4] kg, and body mass index: 22.7 [1.9] kg/m^2^) voluntarily participated in the study. The participants joined an Italian First division team (Serie A), with a minimal soccer background experience of 5 years. Within the season, their typical training volume consisted of four training sessions per week plus one match. Inclusion criteria were: (i) eight hours training per week, (ii) having a current contract with the team, (iii) no history of febrile illness at the time assessments, and (iv) no severe injury over the study. After a detailed explanation of the procedures, the participants signed an informed consent. All research procedures were reviewed and approved by the bioethical committee board of the University of Padova (approval code: HEC-DSB/02-2023) and were conform to the Declaration of Helsinki concerning studies involving human subjects.

### Procedures

Participants came to the training center at 9:00 am refraining stimulant beverages and fasting for at least 2 h. Height and body mass were measured using a mechanical scale with stadiometer (Seca 711, Seca, Hamburg, Deutschland). Body mass index was calculated as total body mass (kilograms) divided by height (meters) squared. The selected anthropometric equations were chosen as the only ones available and appropriate for female athletic subjects [16] and are as follows:

Warner et al. [28]: Fat mass (%) = ((body mass − (8.51 + (0.809 × body mass) - (0.178 × abdominal skinfold) - (0.225 × thigh skinfold)) / body mass) × 100.

Evans et al. [29]: Fat mass (%) = 8.997 + (0.24658 × sum of triceps, abdominal, thigh skinfolds)– (6.343 × sex) - (1.998 × race), where sex is 1 for males and 0 for females and race is 1 for black and 0 for white individuals.

Skinfold thicknesses were measured by a level 3 anthropometrist following the procedures established by the International Society for Advancement of Kinanthropometry (ISAK) [30]. Skinfold thicknesses were measured to the nearest 0.1 mm using a caliper (Harpenden, Baty International Ltd, West Sussex, UK). The technical error of measurement score was within the 5%.

Fat mass was obtained by DXA on a Lunar Prodigy scanner (General Electric, Boston, MA) with enCORE software (v. 17). The scanner was calibrated daily before scanning according to the manufacturer’s indications using a calibration block.

### Statistical analysis

Data were analyzed with Jamovi version 0.9.2.9 (Jamovi project, 2018). The normality of the data was ensured using the Shapiro-Wilk test. To determine whether cross-sectional estimates and changes in fat mass differed between DXA and alternative methods, one-way repeated measures (ANOVA) tests were performed, with method and time as within-subjects factors. When statistically significant effects were observed, follow-up pairwise comparisons were performed using t-tests, with correction for multiple comparisons using the Bonferroni adjustment. Agreement at the group level between methods was also assessed via linear regression analyses, Lin’s concordance correlation coefficient (CCC), and by McBride’s strength concordance (almost perfect > 0.99; substantial > 0.95 to 0.99; moderate = 0.90–­ 0.95; and poor < 0.90) [31]. Agreement at the individual level was evaluated using Bland–Altman analysis and the slope of the trend line was examined to assess proportional bias between methods. Statistical significance was determined using an alpha < 0.05.

## Results

**Fig. 1:**
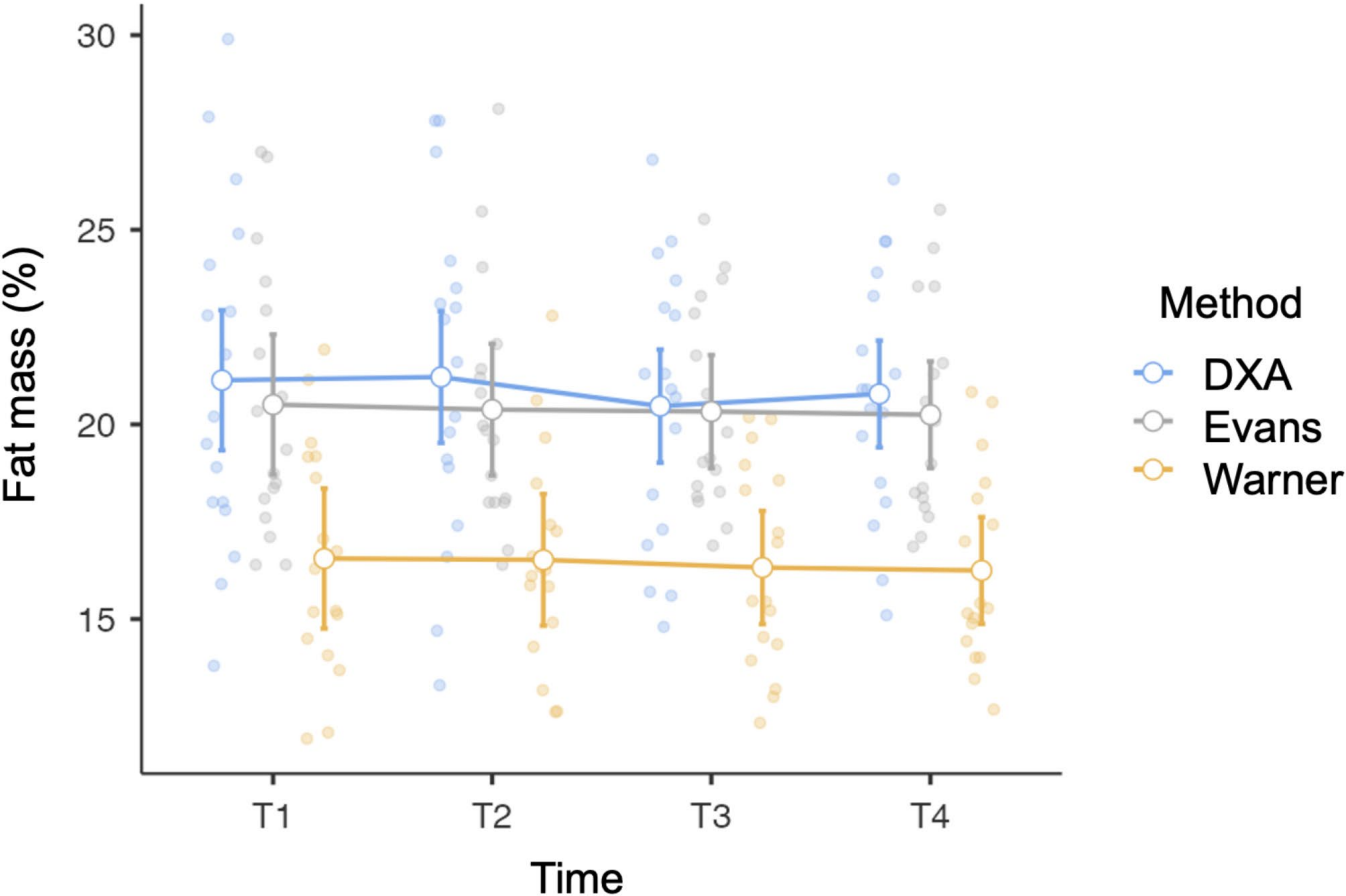
Cross-sectional and longitudinal variability in fat mass percentage at pre-season (T1), at first midseason phase (T2), at second midseason phase (T3), and at the end of the season (T4) in the female soccer players

Figure 1 shows the cross-sectional and longitudinal variability in fat mass percentage among the participants. Raw anthropometric data are reported in Supplementary Table 1.

**Fig. 2:**
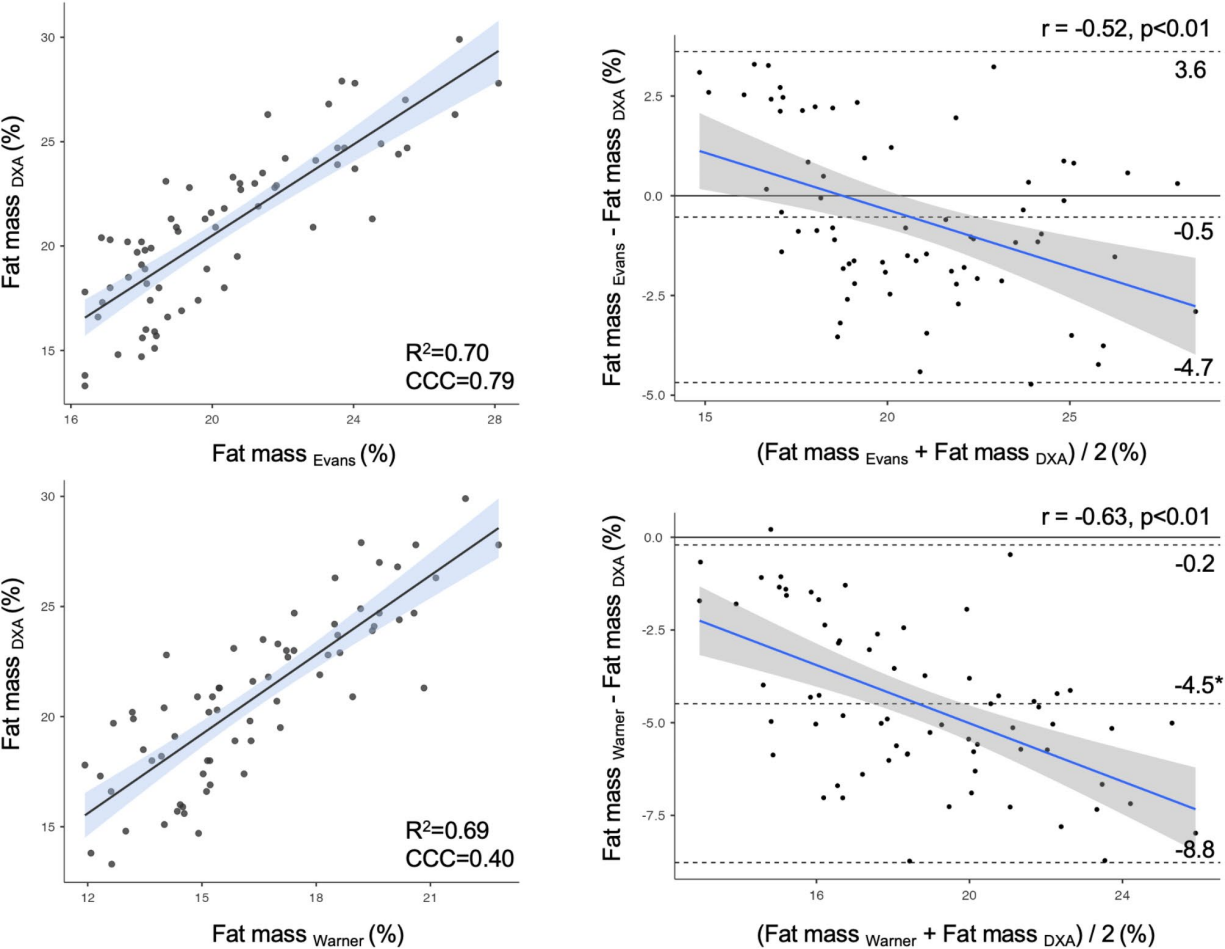
On the left side the scatterplots with the relationship between the predicted and the reference fat mass. On the right side the results of Bland–Altman analyses. *=significant difference; R^2^ = coefficient of determination; CCC = concordance correlation coefficient; r = coefficient of correlation

Regarding the analysis of cross-sectional agreement, since no time effects were observed for DXA (F = 0.89, *p* = 0.45), Evans’s equation (F = 0.12, *p* = 0.98), and Warner’s equation (F = 0.26, *p* = 0.85), the measurements collected at the different time points were considered together for each method, as shown in Fig. 2. Warner’s equation underestimated fat mass compared to DXA (t = 16.9, 95% confidence interval = 3.96–5.02, *p* < 0.001), while both Evans’s and Warner’s equations exhibited a negative trend (*p* < 0.01).

**Fig. 3:**
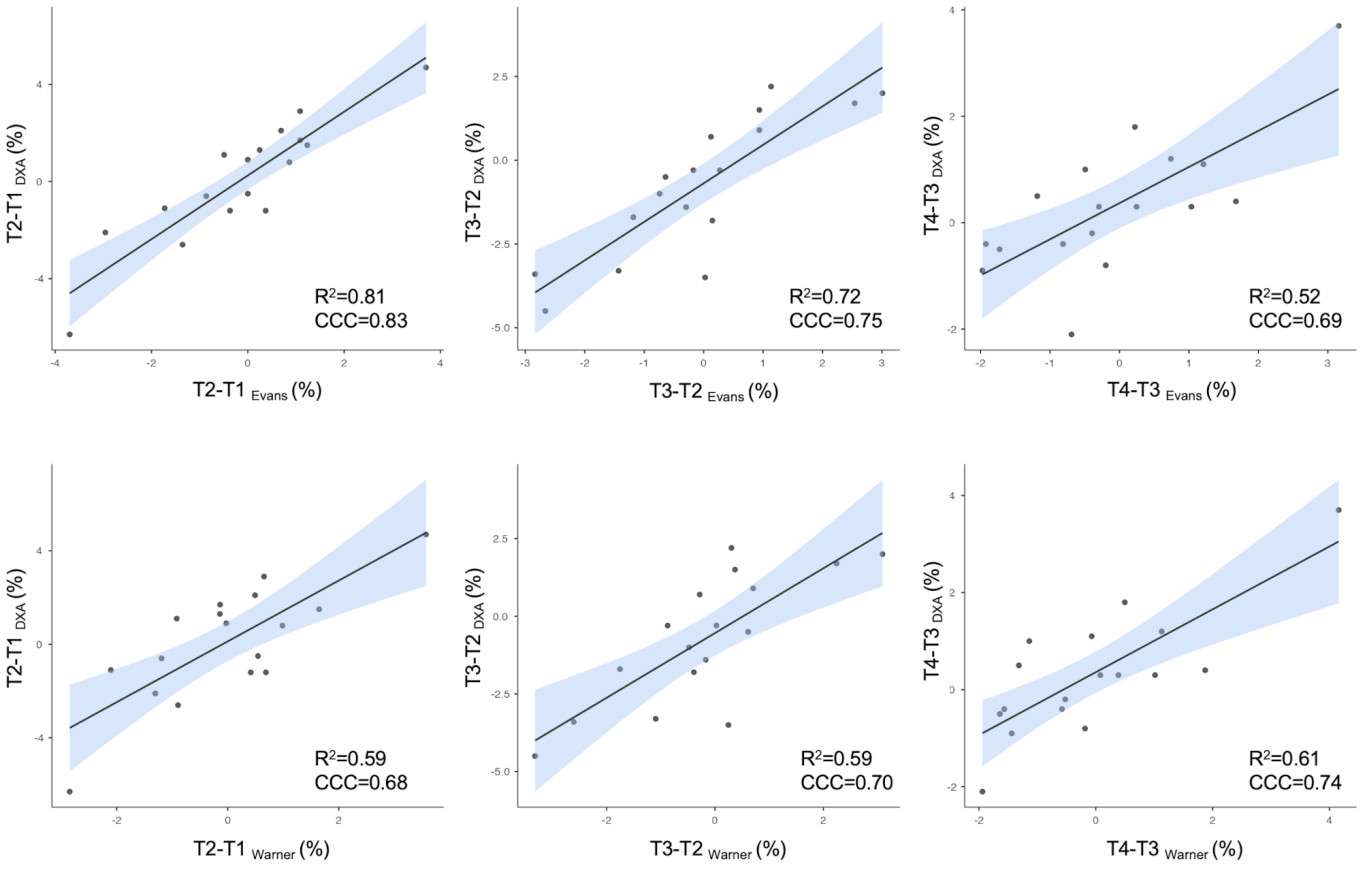
Scatterplots of the relationship between the change in fat mass percentage estimated by the reference method and the predictive equations. R^2^ = coefficient of determination; CCC = concordance correlation coefficient

The results of the longitudinal agreement at the group level are shown in Fig. 3, where changes in fat mass percentage were assessed between T1 and T2, T2 and T3, and T3 and T4.

**Fig. 4:**
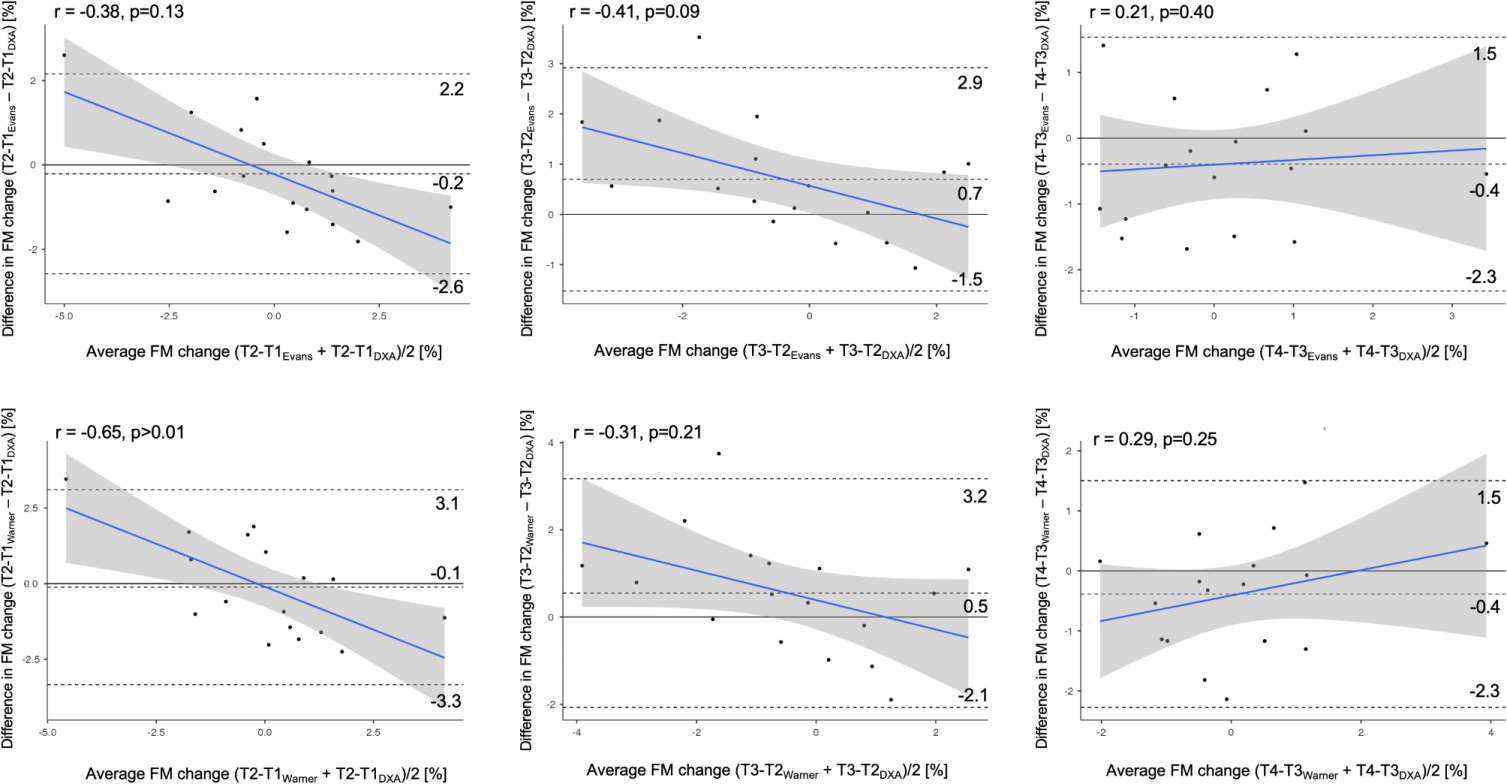
Bland-Altman analysis of the agreement between methods for the change of percentage of fat mass. The middle-dotted line represents the mean differences between the values obtained by the predictive equation and DXA. The upper and lower dotted line represents the 95% limits of agreement (± 1.96 standard deviation). The blue line represents the degree of association between the differences of the methods and the mean of both methods, as illustrated by the coefficient of correlation (r)

The results of the longitudinal agreement at the individual level are shown in Fig. 4, where changes in fat mass percentage were assessed between T1 and T2, T2 and T3, and T3 and T4. Changes from T1 to T4 reported a R^2^ of 0.73 and 0.61, for Evans and Warmer, respectively. No bias was detected between DXA, Evans’s, and Warner’s equations in the changes in fat mass percentage (*p* > 0.008). A positive trend was identified for Warner’s equation in the change in fat mass percentage between T1 and T2 compared to DXA.

## Discussion

Research in sports science underscores the need for more focus on female athletes, who have long been underrepresented in studies on training, nutrition, and body composition [26, 27]. The aim of the present study was to evaluate the accuracy of anthropometric equations selected as the most suitable for estimating body fat in female soccer players. Anthropometric outputs were compared to reference values obtained from DXA throughout a competitive season. Cross-sectional agreement at the group level was identified for the Evans equation, but not at the individual level, where both the Evans and Warner equations tended to overestimate body fat in players with low fat percentages and underestimate it in players with higher fat percentages. Conversely, in the longitudinal analysis, both the Evans and Warner equations proved accurate at the group level, with the Evans equation demonstrating superior performance even at the individual level. Validating anthropometric-based predictive equations specifically for female athletes is crucial, as previous studies have predominantly focused on mixed or male cohorts.

Neither DXA nor the two anthropometric equations detected significant changes in fat mass percentage over the season. For this reason, all values corresponding to the different time points were considered together for each of the three methods in the cross-sectional agreement analysis. The results showed that the Warner equation underestimated fat mass percentage by approximately 4% compared to DXA, whereas no bias was observed between the Evans equation and the fat mass reference. Both equations explained about 70% of the variability, but their agreement fell below optimal levels (< 0.90). Given that both equations were developed using mixed athlete populations, this lack of specificity may account for their limited accuracy at the group level. The Bland-Altman test allows to assess the agreement at the individual level between the alternative methods (e.g., predictive equations) and the reference method (e.g., DXA), quantifying systematic differences and defining limits of agreement to evaluate reliability and consistency. In this case, the scenario worsened, with 95% limits of agreement ranging from − 8.8 to 3.6% and a negative trend observed for both methods. This suggests that neither equation should replace DXA for quantifying fat mass percentage in female soccer players when the goal is to monitor this parameter individually. Similar results have been reported for male soccer players when equations developed from mixed athlete populations were applied [18]. Therefore, soccer-specific equations should be developed to enable more personalized assessments for individual players. Conversely, they can still be useful for evaluating the team’s body composition at specific points during the season, where defining an optimal condition for the entire team is beneficial [32].

The longitudinal analysis showed that the anthropometric equations did not differ from DXA in tracking changes in fat mass percentage. In this context, the Evans equation explained variability ranging from a minimum of approximately 50% to about 80%, though still within a poor agreement range (CCC < 0.90). Nevertheless, its performance was superior to that of the Warner equation, which achieved a maximum R² of 0.61 and a CCC of 0.74 across the different time points. In general, the available solutions for male soccer players demonstrate greater validity and a wider range of options for quantifying fat mass changes during the season [10]. Unfortunately, anthropometric equations specifically designed for female soccer players do not currently exist, and those developed for mixed athlete populations are limited to the two considered in this study [16]. Regarding individual-level agreement, the Evans equation did not exhibit a trend at any of the analyzed time points, with 95% limits of agreement ranging from − 1.5 to 2.9%. This suggests that it represents a viable solution for tracking fat mass changes on an individualized basis. This capability is particularly important for enabling trainers and nutritionists to monitor the body composition of athletes who, for example, join the team mid-season or experience periods of inactivity due to injury, where an increase in body fat could negatively impact health and performance [9, 33].

To date, the best-performing alternative for quantifying fat mass and tracking its changes over time in elite female soccer players, both at the group and individual levels, appears to be the Evans equation [29]. This equation requires only three skinfold measurements (i.e., triceps, abdomen, and thigh) along with sex and ethnicity-related codes. While our findings suggest it as the best available solution, new formulas are needed to fully meet the accuracy requirements for monitoring body fat in high-level female soccer players. To achieve this, future formulas should incorporate additional skinfold sites and anthropometric measurements, such as body circumferences. Such advancements would particularly benefit sub-elite teams, where limited resources often preclude the use of DXA for body composition assessments. Accurate fat mass evaluations could help identify players who need to adjust their dietary habits or undergo tailored training loads during specific phases of the season [34]. This is especially important in soccer, unlike other sports (e.g., rugby or American football), where certain positions (e.g., defensive linemen) may benefit from slightly higher fat mass [13]. While this article provides valuable insights for practitioners, certain limitations should be acknowledged. For example, the results of this study are not generalizable to athletes in other female sports or to adolescent soccer players. Additionally, the reference method used to quantify fat mass was not the gold standard for this purpose. A four-compartment model, which integrates multiple techniques such as DXA, air displacement plethysmography, and dilution techniques, would provide a more accurate assessment. This model accounts for the variability in hydration levels of lean soft mass, offering a more precise evaluation of body composition compared to simpler methods [35]. Furthermore, future studies should focus on evaluating other body composition parameters and investigating how their variations may contribute to improved athletic performance in soccer. Lastly, the 95% limits of agreement ranged from − 3.3 to 3.2%, which might compromise the ability to make practical decisions in training interventions. Although an elite population was considered, a larger sample size should be employed in future longitudinal validation studies.

## Conclusions

This study identifies the Evans equation as the best available alternative for quantifying fat mass and monitoring its changes during the competitive season in female soccer players. This equation requires the measurement of only three anthropometric parameters (triceps, abdominal, and thigh skinfolds), making it a more cost-effective, faster, and generally user-friendly solution compared to DXA. However, given that its predictive accuracy at both group and individual levels could be improved, the development of soccer-specific anthropometric equations tailored to female players is warranted.

## Electronic supplementary material

Below is the link to the electronic supplementary material.

Supplementary Material 1

## Data Availability

Data are available from the corresponding author on request.
