# Supplementary material for: Accuracy of anthropometric-based predictive equations for tracking fat mass over a competitive season in elite female soccer players: a validation study
Source: BMC Sports Sci Med Rehabil. 2025 Apr 3;17:68. doi: 10.1186/s13102-025-01115-4 (PMC11967043; doi:10.1186/s13102-025-01115-4)
Supplement: Supplementary file 1 — Supplementary Material 1 [file 13102_2025_1115_MOESM1_ESM.docx]

| **Supplementary table 1**. Skinfold thicknesses (mean ± standard deviation) measured across the competitive period. | | | | |
| --- | --- | --- | --- | --- |
|  | T1 | T2 | T3 | T4 |
| Triceps (mm) | 13.9 ± 4.3 | 13.6 ± 4.4 | 14.1 ± 3.6 | 14.1 ± 4.2 |
| Abdominal (mm) | 15.4 ± 6.9 | 15.2 ± 6.0 | 13.9 ± 5.7 | 14.2 ± 5.8 |
| Thigh (mm) | 18.9 ± 4.5 | 18.7 ± 4.7 | 19.4 ± 4.1 | 18.7 ± 4.0 |
